# Carbon monoxide attenuates amyloidogenesis via down‐regulation of NF‐κB‐mediated BACE1 gene expression

**DOI:** 10.1111/acel.12864

**Published:** 2018-11-09

**Authors:** Hyo Jeong Kim, Yeonsoo Joe, Yingqing Chen, Gyu Hwan Park, Uh‐Hyun Kim, Hun Taeg Chung

**Affiliations:** ^1^ Meta‐Inflammation Research Institute of Basic Research, School of Biological Sciences University of Ulsan Ulsan South Korea; ^2^ College of Pharmacy, Research Institute of Pharmaceutical Sciences Kyungpook National University Daegu South Korea; ^3^ National Creative Research Laboratory for Ca^2+^ Signaling Network, Medical School Chonbuk National University Jeonju South Korea

**Keywords:** 27‐hydroxycholesterol, Alzheimer’s disease, BACE1, carbon monoxide, NF‐κB, SIRT1

## Abstract

Amyloid‐β (Aβ) peptides, the major constituent of plaques, are generated by sequential proteolytic cleavage of the amyloid precursor protein (APP) via β‐secretase (BACE1) and the γ‐secretase complex. It has been proposed that the abnormal secretion and accumulation of Aβ are the initial causative events in the development of Alzheimer's disease (AD). Drugs modulating this pathway could be used for AD treatment. Previous studies indicated that carbon monoxide (CO), a product of heme oxygenase (HO)‐1, protects against Aβ‐induced toxicity and promotes neuroprotection. However, the mechanism underlying the mitigative effect of CO on Aβ levels and BACE1 expression is unclear. Here, we show that CO modulates cleavage of APP and Aβ production by decreasing BACE1 expression in vivo and in vitro. CO reduces Aβ levels and improves memory deficits in AD transgenic mice. The regulation of BACE1 expression by CO is dependent on nuclear factor‐kappa B (NF‐κB). Consistent with the negative role of SIRT1 in the NF‐κB activity, CO fails to evoke significant decrease in BACE1 expression in the presence of the SIRT1 inhibitor. Furthermore, CO attenuates elevation of BACE1 level in brains of 3xTg‐AD mouse model as well as mice fed high‐fat, high‐cholesterol diets. CO reduces the NF‐κB‐mediated transcription of BACE1 induced by the cholesterol oxidation product 27‐hydroxycholesterol or hydrogen peroxide. These data suggest that CO reduces the NF‐κB‐mediated BACE1 transcription and consequently decreases Aβ production. Our study provides novel mechanisms by which CO reduces BACE1 expression and Aβ production and may be an effective agent for AD treatment.

## 1 INTRODUCTION

Alzheimer's disease (AD) is the most prevalent form of dementia, which is a kind of central nervous system disease characterized by progressive memory loss and cognitive decline. Almost 50 million people worldwide are living with dementia, and about 60%–70% of them is known to be AD patients. Pathologically, AD shows three major features of amyloid plaques, neurofibrillary tangles, and neuronal loss. Amyloid‐β (Aβ) peptides, the major component of plaques, are produced from sequential proteolytic cleavage of the amyloid precursor protein (APP) via β‐secretase (BACE1) and γ‐secretase (Hardy & Selkoe, 2002) (also called amyloidogenic pathway). The BACE1 expression and activity is elevated in the brains of patients with sporadic AD (Yang et al., 2003), suggesting a causative role of BACE1 in AD.

Dysregulation of BACE1 expression at transcription and translation levels might be involved in AD pathogenesis. Numerous studies have demonstrated that oxidative stress‐induced BACE1 gene expression and activity is regulated by a number of redox‐sensitive transcription factors such as HIF (Sun et al., 2006), STAT1 (Cho et al., 2009), and NF‐κB (Bourne et al., 2007). Metabolic risk factors such as hypercholesterolemia, hypertension, and diabetes have been shown to increase the risk of AD (Bhat, 2010; Thirumangalakudi et al., 2008). Hypercholesterolemia was shown to increase neuroinflammatory changes in the brain via oxidative stress. High‐fat diet‐related increase in oxidative stress increases inflammatory cytokines in the hippocampus and cortex (Alzoubi, Khabour, Salah, & Hasan, 2013; Liu et al., 2014; Thirumangalakudi et al., 2008; Xia et al., 2015).

BACE1 transcription is upregulated by cholesterol oxidized metabolite 27‐hydroxycholesterol (27‐OHC)‐activated NF‐κB (Marwarha, Raza, Prasanthi, & Ghribi, 2013) and suppressed by metabolic regulatory pathway of SIRT1‐PGC1a (Wang et al., 2013). It was reported that both NF‐κB and BACE1 levels are increased in sporadic AD patients, and NF‐κB enhances BACE1 gene expression and APP processing (Chen et al., 2012). NF‐κB is involved in the gene expression of multiple inflammatory pathways and that protective drugs could exert multiple anti‐inflammatory actions as downregulating factors of NF‐κB (Piva, Belardo, & Santoro, 2006).

Drugs targeting BACE1 are believed to be one of the most promising strategies for AD treatment. While BACE1 overexpression increases Aβ formation in APP transgenic mice (Bodendorf et al., 2002), BACE1‐knockout mice show abolished Aβ generation (Luo et al., 2001). BACE1 suppression by RNA interference in primary cortical neurons reduces APP beta‐c‐terminal fragments (β‐CTFs) and Aβ production (Kao, Krichevsky, Kosik, & Tsai, 2004), and deficiency of BACE1 gene rescues memory deficits and cholinergic dysfunction in the Tg2576 AD model (Ohno et al., 2004). Oral administration of a potent and selective non‐peptidic BACE1 inhibitor decreases β‐cleavage and Aβ production in vivo (Hussain et al., 2007).

Sirtuin 1 (SIRT1) is the NAD‐dependent protein deacetylase implicated in diverse cellular processes, including metabolism, development, stress response, neurogenesis, inflammation, and apoptosis (Kim et al., 2007; Michan & Sinclair, 2007; Morris, Lin, Thompson, & Perez‐Pinzon, 2011). SIRT1 attenuates Aβ toxicity in primary neuronal cells by the inhibition of NF‐κB signaling (Chen et al., 2005). Leptin‐activated SIRT1 reduces Aβ level by attenuating NF‐κB‐mediated BACE1 expression (Marwarha, Raza, Meiers, & Ghribi, 2014). Overexpression of SIRT1 renders a neuroprotective effect in models of AD (Kim et al., 2007). Moreover, SIRT1 activators, such as resveratrol, could exert protective effects in experimental model of Alzheimer disease. The protective effects could be partially related to down‐regulation of NF‐κB (Buhrmann, Shayan, Popper, Goel, & Shakibaei, 2016; Chiavaroli et al., 2010).

Carbon monoxide (CO), a reaction product of heme oxygenase (HO) activity, can exert potent anti‐inflammatory, anti‐proliferative, and anti‐apoptotic effects in vitro and in vivo and thereby mimic the cytoprotective effect of HO‐1 (Brouard et al., 2000; Otterbein et al., 2000, 2003 ). HO‐1 protects against amyloid‐β‐induced toxicity via CO production (Hettiarachchi et al., 2014). CORM, a carbon monoxide‐releasing molecule, promotes neuroprotection in brain injury model (Yabluchanskiy et al., 2012). In addition, CO offers neuroprotection from hippocampal cell damage through the PERK‐activated ER stress pathway (Han et al., 2015).

On the basis of these observations, we aimed to define potential modulation of BACE1 expression by CO could play a role in ameliorating AD neuropathology and identified its underlying mechanism. We found that CO not only alleviates memory deficits in AD transgenic mice, but also inhibits APP cleavage by reducing BACE1 expression level via the increase in SIRT1 expression and inhibition of NF‐κB signaling. Thus, we show that CO regulates Aβ levels and provide the molecular mechanisms involved in the suppression of Aβ formation. Our results provide beneficial effect of CO as a possible therapeutic agent for AD.

## 2 RESULTS

### 2.1 CO reduces cortical BACE1 expression in 3xTg and HFC diet mice

**Figure 1:**
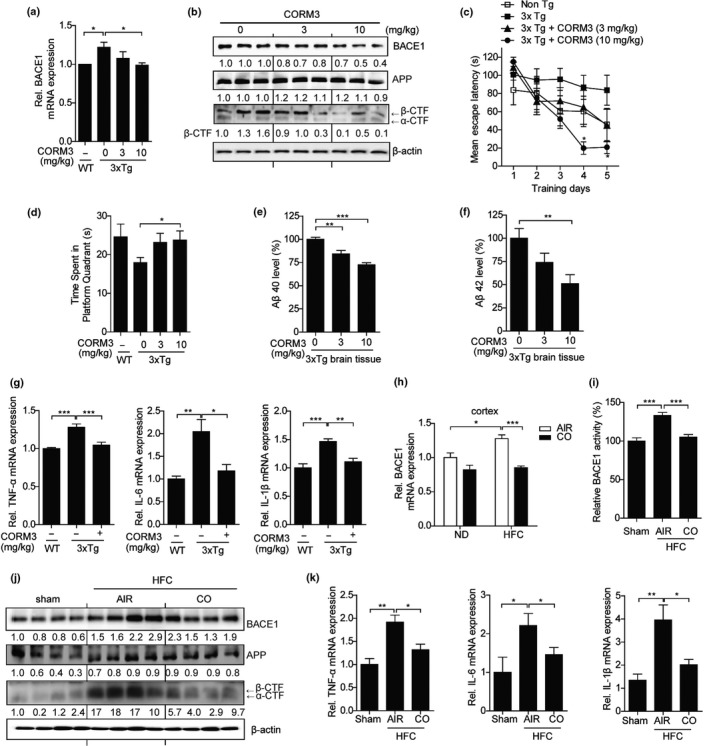
CO reduces cortical BACE1 expression in 3xTg and HFC diet mice. (a–g) Wild‐type (Non‐Tg) or 3xTg‐AD mice were intraperitoneally injected daily for 1 month with CORM3 (3 or 10 mg/kg) or a vehicle solution and subjected to the Morris water maze test. (a) The expression of BACE1 in CORM3‐treated 3xTg‐AD and Non‐Tg brains was determined by qRT–PCR. Data were represented as mean ± *SEM* four technical replicates, *p < 0.05. (b) Protein levels in CORM3 administrated 3xTg‐AD and non‐transgenic mouse brains were measured by immunoblotting with anti‐BACE1 antibody, and anti‐APP antibody (A8717) for detecting full‐length APP and CTFs. β‐actin was used as an internal control. *N* = two independent experiments. (c) Morris water maze test was performed to analyze the effect of CO on impairment of learning and memory in 3xTg‐AD mice as indicated by reduced performance to find the hidden platform (non‐Tg with vehicle, *n* = 5; 3xTg‐AD with vehicle, *n* = 7; 3xTg‐AD with CORM 3 mg/kg, *n* = 5; 3xTg‐AD with CORM 10 mg/kg, *n* = 4). Data were shown as mean ± *SEM*. *p < 0.05. (d) Probe test was performed on the last day after 5 days of training phase. Data were shown as mean ± *SEM* of three independent experiments. *p < 0.05. (e, f) Aβ40 and Aβ42 in the whole brain lysates were measured by ELISA. (g) The gene expression of TNF‐α, IL‐6, and IL‐1β were determined by quantitative RT–PCR. Data were represented as mean ± *SEM* from four technical replicates, *p < 0.05, **p < 0.01. ***p < 0.001. (h–k) HFC or control chow diets fed mice were inhaled with air or CO. (h) Cortical BACE1 mRNA levels were determined by qRT–PCR. Data were represented as mean ± *SEM* from four technical replicates, *p < 0.05, ***p < 0.001. (i) BACE1 activity was evaluated using brain lysates by a fluorescent‐based β‐secretase activity assay kit. (j) Cortical proteins were analyzed by immunoblotting with anti‐BACE1 antibody, and anti‐APP antibody (A8717) for detecting full‐length APP and CTFs. β‐actin was used as an internal control. *N* = two independent experiments. (k) The gene expression of TNF‐α, IL‐6, and IL‐1β were determined by quantitative RT–PCR. Data were represented as mean ± *SEM* three technical replicates. *p < 0.05, **p < 0.01. All results are determined by one‐way or two‐way analysis of variance (ANOVA) followed by Bonferroni post hoc test and are representative of two independent experiments

We tried to know the effect of CO on BACE1 expression in the brain of triple‐transgenic 3xTg‐AD mice. Intraperitoneal injection of CORM3 significantly reduced BACE1 mRNA and protein expression in the cortex of 3xTg mice compared to the non‐treated group (Figure 1a,b). Furthermore, CO significantly decreased the level of the β‐CTFs, which is the main product of β‐secretase (Figure 1b). As shown in Figure 1c, 3xTg mice took a longer time to find the platform compared to the wild‐type mice, whereas CO‐treated 3xTg mice significantly decreased the escape latency. The escape latency of CORM3‐treated mice on the fourth and fifth days of the hidden platform test was shorter than that of vehicle‐treated mice. On the last day, the probe test showed that CORM‐treated mice spent more time in that quadrant of Morris water maze (MWM) test where the hidden platform was placed during training (Figure 1d). Furthermore, the levels of Aβ40 and Aβ42 were dramatically reduced in CORM‐treated mice as compared with the vehicle‐treated controls (Figure 1e,f). To confirm the effect of CO on amyloid plaque pathology, we performed immunohistochemistry for amyloid plaques. CORM3 treatment markedly reduced amyloid plaque formation in the 3xTg mouse brain (Supporting Information Figure S1A). In addition, CORM3 treatment decreased the expression of the pro‐inflammatory cytokines, TNF‐α, IL‐6, and IL‐1β, in 3xTg mouse brain (Figure 1g).

In a previous study, young WT C57BL/6 mice consuming a high‐fat, high‐cholesterol (HFC) diet showed neuroinflammation and cognitive deficit as well as up‐regulation of BACE1 transcription (Thirumangalakudi et al., 2008; Wang et al., 2013). Therefore, we examined the effects of CO on the elevated BACE1 expression in HFC mice. As shown in Figure 1h–J, the levels of BACE1 mRNA, protein expression and activity were increased in the mouse cortex after HFC feeding. Inhalation of CO for 2 weeks (2 hr per day) showed a significant reduction in the levels of BACE1 expression, activity, and β‐CTFs relative to HFC mice inhaled air (Figure 1h–J), but had no significant effect on APP expression (Figure 1j and Supporting Information Figure S1B). In addition, quantitative RT–PCR analysis showed a dramatic decrease in the expression of TNF‐α, IL‐6, and IL‐1β in CO‐inhaled mice (Figure 1k). These data suggest that CO suppresses BACE1 expression and attenuates BACE1‐mediated APP processing in 3xTg and HFC mouse brains.

### 2.2 CO reduces BACE1 expression and activity

**Figure 2:**
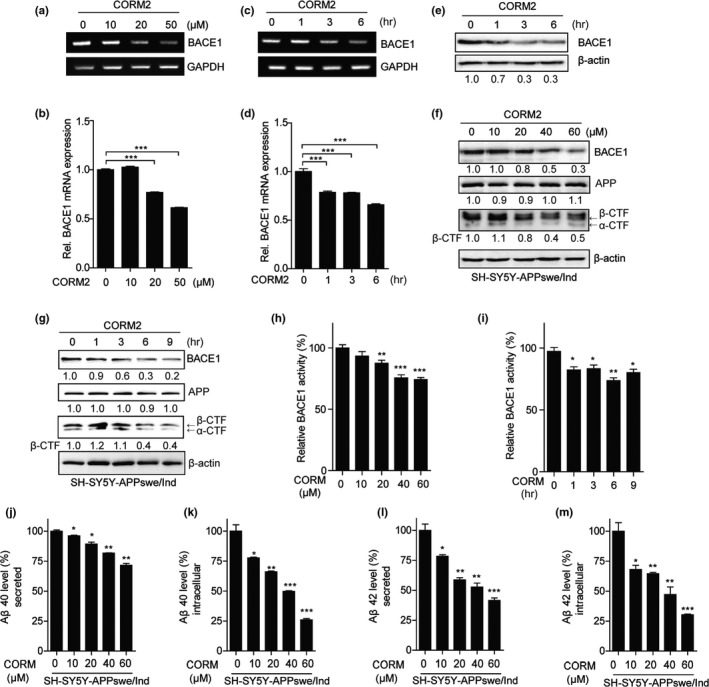
CO reduces BACE1 expression and activity in SH‐SY5Y cells. (a–d) Human neuroblastoma, SH‐SY5Y cells were treated with CORM2 at the indicated doses for 6 hr (a, b), or 20 μM CORM2 for the indicated times (c, d). Expression of BACE1 mRNA was examined by semiquantitative RT–PCR (a, c) and qRT–PCR (b, d). Data were represented as mean ± *SEM* from three technical replicates, ***p < 0.001 vs. untreated control, via one‐way ANOVA followed by Tukey's test. (e) SH‐SY5Y cells were treated with CORM2 for 6 hr. Protein levels of BACE1 were performed by immunoblotting. *N* = three independent experiments. (f–i) SH‐SY5Y cells were transfected with pCAX APP Swe/Ind constructs, expressing human amyloid precursor protein (APP) 695 Swedish mutation. After 24 hr, cells were treated with CORM2 at the indicated doses for 6 hr (f, h) or 20 μM CORM2 for the indicated times (g, i). (f, g) Protein levels were analyzed by immunoblotting with BACE1 antibody and APP antibody (A8717) for detecting full‐length APP and CTFs. *N* = three independent experiments. (h, i) BACE1 activity was performed by a fluorescent‐based β‐secretase activity assay kit. (j–m) SH‐SY5Y cells were transfected with pCAX APP Swe/Ind constructs, expressing human amyloid precursor protein (APP) 695 Swedish mutation. After 24 hr, cells were treated with CORM2 at the indicated doses for 6 hr. The levels of secreted or intracellular Aβ40 (j, k) and Aβ42 (l, m) were measured by ELISA. Data were represented as mean ± *SEM* from three technical replicates, *p < 0.05, **p < 0.01, ***p < 0.001 vs. untreated control, via one‐way ANOVA followed by Tukey's test (h–m). Results are representative of three independent experiments

Given the suppressive effects of CO in vivo on the BACE1 expression, we asked whether CORM2 would also suppress BACE1 gene expression in vitro. To examine the effects of CO on BACE1 expression and its activity, we treated SH‐SY5Y cells with CORM2. CORM2 significantly decreased the levels of BACE1 mRNA and protein in a dose‐ and time‐dependent manner (Figure 2a–g). BACE1 mRNA levels began to decrease at 20 μM and 3 hr, respectively (Figure 2a–d). Especially, CORM2 reduced the levels of β‐CTFs in SH‐SY5Y‐APPswe/Ind or N2aswe cells, overexpressing Swedish mutant APP (Figure 2f,g and Supporting Information Figure S1C). The BACE1 protein expression was decreased at 3 hr after the treatment of CORM2 (Figure 2e,g), and the levels of β‐CTFs were reduced at 6 hr (Figure 2g). Because BACE1‐mediated APP processing is the first step leading to Aβ generation, we asked whether CO inhibits BACE1 activity. As shown in Figure 2h,i, CO dose‐ and time‐dependently reduced the BACE1 activity. Next, to determine whether CO can affect Aβ generation, we measured Aβ40 and Aβ42 levels in APPswe/Ind‐overexpressed SH‐SY5Y cells. CO inhibited the intracellular and secreted Aβ40 and Aβ42 levels in a dose‐dependent manner (Figure 2j–m). These results indicated that CO blocks Aβ production at the level of BACE1. These data demonstrate that, consistent with in vivo results, CO specifically decreases BACE1 gene expression and its β‐secretase activity in vitro.

### 2.3 Regulation of BACE1 expression by CO is mediated by SIRT1

**Figure 3:**
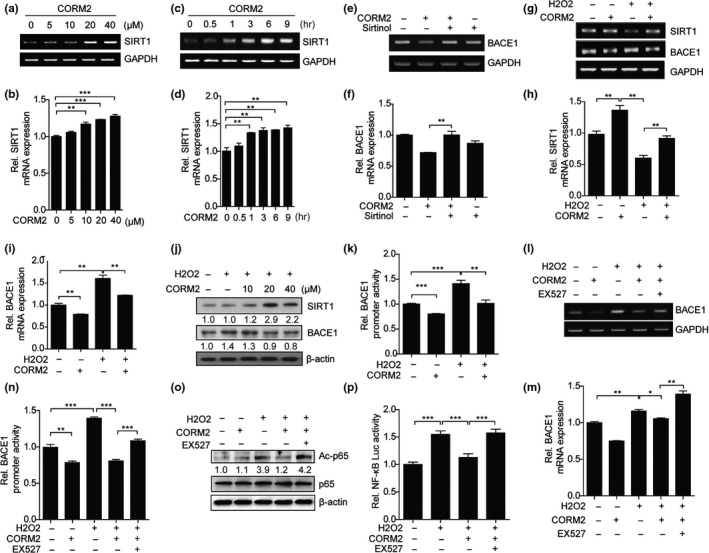
CO‐induced SIRT1 reduces oxidative stress‐induced BACE1 expression through negative regulation of NF‐κB transcriptional activity. (a–d) SH‐SY5Y cells were treated with CORM2 at the indicated doses for 6 hr (a, b) and 20 μM CORM2 for the indicated times (c, d). SIRT1 mRNA levels were examined by semiquantitative RT–PCR (a, c) and qRT–PCR (b, d). Data were represented as mean ± *SEM* from three technical replicates, **p < 0.01, ***p < 0.001, via one‐way ANOVA followed by Tukey's test. Results are representative of three independent experiments. (e, f) SH‐SY5Y cells were pre‐incubated with 400 μM sirtinol for 24 hr and treated with 20 μM of CORM2 for 6 hr. The levels of BACE1 mRNA expression were determined by semiquantitative RT–PCR (e) and qRT–PCR (f). (g–i) SH‐SY5Y cells were pretreated with CORM2 and incubated with 200 μM of H_2_O_2_ for 3 hr. The mRNA levels of SIRT1 and BACE1 were measured by semiquantitative RT–PCR (g) and qRT–PCR (h, i). (j) SH‐SY5Y cells were incubated with 200 μM of H_2_O_2_ for 3 hr in the presence of CORM2 at the indicated doses. The protein levels of SIRT1 and BACE1 were performed by immunoblotting. *N* = three independent experiments. (k) SH‐SY5Y cells were co‐transfected with BACE1P‐2.5 constructs or pGL3 basic vector and pRL‐SV40 plasmid for 24 hr, and then, cells were treated with H_2_O_2_ (200 μM) in the presence of CORM2 at the indicated doses. After 12 hr, luciferase activity was measured. (l, m) SH‐SY5Y cells were pretreated with or without the SIRT1 inhibitor (EX527; 40 μM) for 1 hr, followed by treatment with CORM2 (20 μM) alone or in combination for 1 hr and harvest after incubating with H_2_O_2_ (200 μM) for 6 hr. The levels of BACE1 mRNA expression were determined by semiquantitative RT–PCR (l) and qRT–PCR (m). (n) BACE1P‐2.5 and pRL‐SV40 plasmid were co‐transfected into SH‐SY5Y cells, and then, cells were treated with H_2_O_2_ (200 μM) in the presence of CORM2 (20 μM) and EX527 (40 μM). After 12 hr, luciferase activity was measured. (o) SH‐SY5Y cells were pretreated with or without the SIRT1 inhibitor (EX527; 40 μM) for 1 hr, followed by treatment with CORM2 (20 μM) alone or in combination for 1 hr and harvest after incubating with H_2_O_2_ (200 μM) for 1 hr. The protein levels of Ac‐p65 and p65 were measured by immunoblotting. *N* = three independent experiments. (p) SH‐SY5Y cells were transfected with NF‐κB responsive element reporter constructs (NF‐κB‐Luc) and pRL‐SV40 plasmid for 48 hr. Cells were pretreated with CORM2 and stimulated with 200 μM of H_2_O_2_. After 6 hr, luciferase activity was measured. Data (f, h, i, k, n, p, m) were represented as mean ± *SEM* from three technical replicates, *p < 0.05, **p < 0.01, ***p < 0.001 via two‐tailed Student's *t* test. Results are representative of three independent experiments

BACE1 was reported to be downregulated by SIRT1 (Marwarha et al., 2014; Wang et al., 2013). To know whether SIRT1 mediates CO‐induced suppression of BACE1 expression, we measured the effects of CO on BACE1 expression in the presence of sirtinol, a SIRT1 inhibitor. From this assay, we confirmed that CORM2 treatment dose‐ or time‐dependently increased SIRT1 expression in SH‐SY5Y cells (Figure 3a–d). CORM2 treatment decreased BACE1 expression, but it lost the suppressive effect of CO on the BACE1 expression in the presence of sirtinol (Figure 3e–f). Since oxidative stress causes an increased amyloid‐beta and BACE1 expression in SH‐SY5Y cells (Gu, Sun, Li, Wu, & Li, 2013), we treated the cells with CORM2 in the presence of H_2_O_2_. Pretreatment the cells with CORM2 increased the SIRT1 expression, resulting in the suppression of BACE1 expression (Figure 3g–j and Supporting Information Figure S1D–G). It has been reported that H_2_O_2_ induces APP expression and therefore enhances Aβ production (Frederikse, Garland, Zigler, & Piatigorsky, 1996). To exclude the possibility that the suppressive effects of CO reduce APP mRNA expression, we measured the effects of CO on APP mRNA expression in H_2_O_2_‐treated cells and found no differences between CO‐treated and CO‐untreated cells (Supporting Information Figure S1H). To investigate the mechanism underlying the effects of CO on BACE1 gene expression at the transcriptional levels, we performed a BACE1 gene promoter assay using human BACE1 gene promoter constructs. Regions of the BACE1 promoter from −2,575 to +227 (BACE1P‐2.5) were inserted into promoterless vector pGL3‐basic upstream of firefly luciferase reporter gene. To know whether CO regulates BACE1 gene transcription, we transfected SH‐SY5Y cells with BACE1P‐2.5 constructs and then treated them with H_2_O_2_ in the presence of CORM2. CO significantly decreases the promoter activity of BACE1P‐2.5 in SH‐SY5Y cells (Figure 3k). Furthermore, treatment of the cells with SIRT1 inhibitor, EX527, abolished the suppressive effects of CO on BACE1 expression and promoter activity (Figure 3l–n). These data indicate that CO‐induced SIRT1 negatively regulates BACE1 expression.

### 2.4 SIRT1 induced by CO reduces oxidative stress‐induced BACE1 expression through suppression of NF‐κB transcriptional activity

Multiple lines of evidence have established a positive role of the transcription factor NF‐κB in BACE1 transcription (Bourne et al., 2007; Buggia‐Prevot, Sevalle, Rossner, & Checler, 2008; Guglielmotto et al., 2012; Ly et al., 2013). SIRT1 has been demonstrated to inhibit NF‐κB‐mediated transcription by deacetylating the Lys310 residue in the p65 subunit (Yeung et al., 2004). We hypothesized that CO may regulate BACE1 expression by inhibiting NF‐κB transcriptional activity via the SIRT1 activation. We tried to know the acetylation status of the p65 subunit of NF‐κB in cells treated with CORM2 in the presence and absence of the H_2_O_2_. As shown in Figure 3o, CORM2 treatment reduced the levels of the acetylated‐Lys310 p65 subunit of NF‐κB. Additionally, CORM2 treatment failed to reduce the acetylated p65 induced by H_2_O_2_ in EX527‐co‐treated cells. To make sure the effects of CO on NF‐κB‐mediated BACE1 expression as well as to elucidate the involvement of SIRT1 in mediating these effects, a dual luciferase assay was performed using NF‐κB reporter constructs. CO‐attenuated NF‐kB transcriptional activity was induced by H_2_O_2_ (Figure 3p and Supporting Information Figure S1I). These results suggest that CO suppresses the NF‐κB transcriptional activity through SIRT1 induction in oxidative stress conditions.

### 2.5 CO decreases 27‐OHC‐induced BACE1 transcription

**Figure 4:**
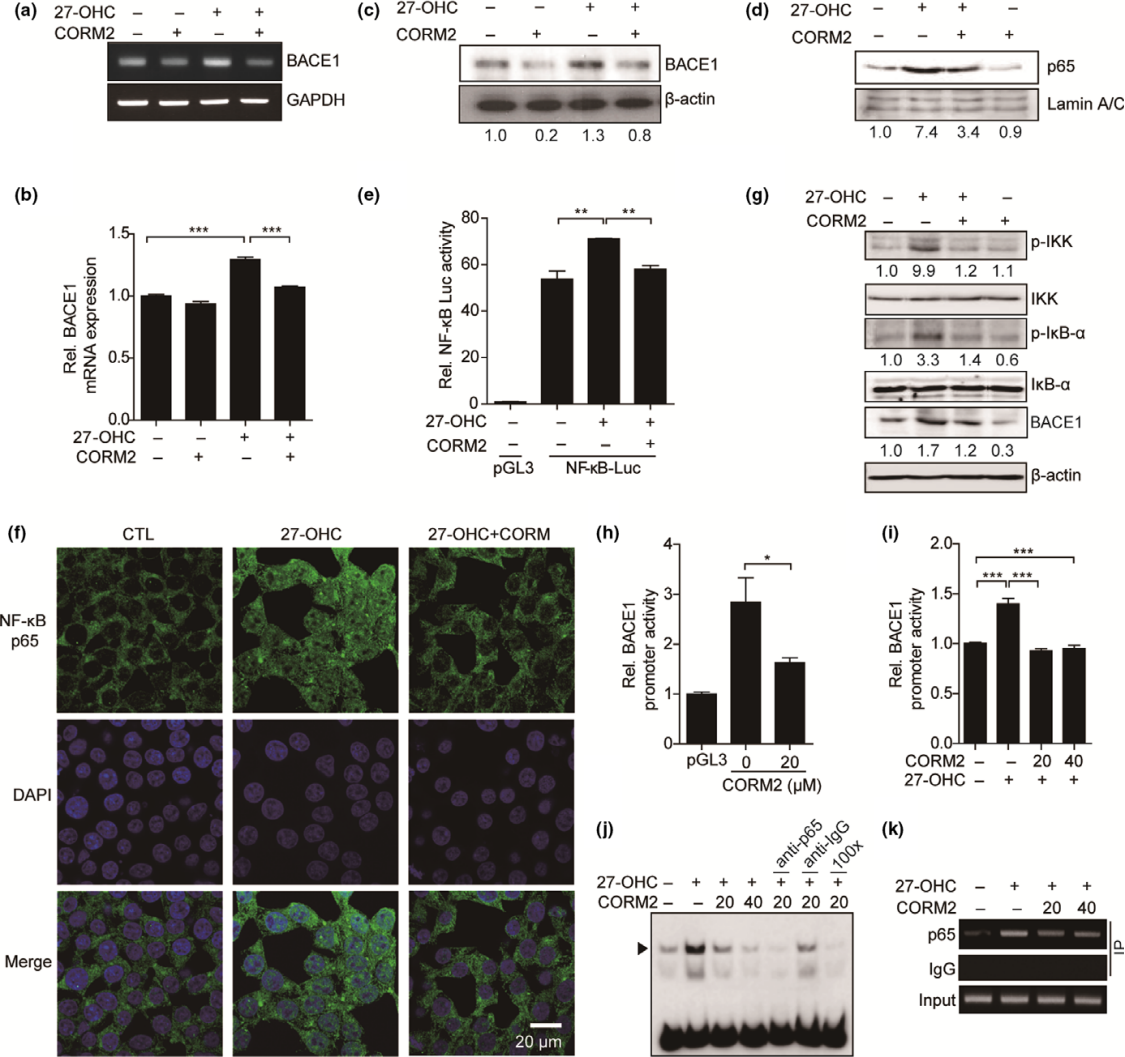
CO suppresses NF‐κB‐mediated BACE1 transcription induced by 27‐OHC in SH‐SY5Y cells. (a–d) SH‐SY5Y cells were pre‐incubated with 20 μM CORM2 for 30 min and treated with 5 μM of 27‐OHC. (a, b) The BACE1 mRNA levels were determined by semiquantitative RT‐PCR (a) and qRT‐PCR (b). Data were represented as mean ± *SEM* from three technical replicates, ***p < 0.001 via two‐tailed Student's *t* test. Results are representative of three independent experiments. (c) The protein levels of BACE1 were measured by immunoblotting. *N* = three independent experiments. (d) The levels of NF‐κB p65 in the nuclear fraction were analyzed by immunoblotting. Lamin A/C was used as nuclear control. *N* = three independent experiments. (e) SH‐SY5Y cells were co‐transfected with NF‐κB responsive element reporter constructs (NF‐κB‐luc) and pRL‐SV40 plasmid for 48 hr. Cells were treated with 5 μM of 27‐OHC in the presence or absence of CORM2. After 6 hr, luciferase activity was measured. (f, g) SH‐SY5Y cells were exposed to 5 μM of 27‐OHC for 12 hr in the presence of or in the absence of 20 μM CORM2. (f) NF‐κB p65 unit was stained in green, and nuclei were visualized by DAPI counterstain. Scale bar = 20 μm. (g) Phosphorylated IKK (p‐IKK), IKK, phosphorylated IκB‐α (p‐IκB‐α) and IκB‐α were detected by immunoblotting. *N* = three independent experiments. (h) SH‐SY5Y cells were transfected with BACE1P‐2.5 constructs or pGL3 basic vector and pRL‐SV40 plasmid. After 24 hr, cells were exposed to 20 μM CORM2 for 6 hr. Cells were lysed and performed luciferase assay. (i) SH‐SY5Y cells were co‐transfected with BACE1P‐2.5 constructs and pRL‐SV40 plasmid. After 24 hr, cells were treated with 27‐OHC (5 μM) in the presence or absence of CORM2 at the indicated doses. (j, k) SH‐SY5Y cells were pre‐incubated with CORM2 (20 or 40 μM) for 30 min and stimulated with 5 μM of 27‐OHC for 6 hr. (j) Nuclear extracts were prepared for EMSA and incubated with biotin‐labeled probes containing NF‐κB responsive element of the BACE1 promoter (BACE1P‐NF‐κB RE). To identify immunoreactivity of NF‐κB p65 in the DNA–protein complexes, nuclear extracts were incubated with anti‐p65 antibody or normal rabbit IgG before the addition of the biotin‐labeled probes. Competition experiments were performed using a 100‐fold excess of unlabeled BACE1P‐NF‐κB RE oligonucleotide. Arrows indicate the DNA–protein complexes. (k) ChIP analysis was performed to confirm the NF‐κB p65 recruitment to the BACE1 promoter. Formaldehyde cross‐linked chromatin was incubated with anti‐p65 antibody or normal rabbit IgG. Total input DNA was used as positive control. Data were represented as mean ± *SEM* from three technical replicates, *p < 0.05, **p < 0.01, ***p < 0.001, via two‐tailed Student's *t* test (e, h, i). Results are representative of three independent experiments

Previous studies demonstrated that 27‐OHC mediates negative effects of dietary cholesterol on cognitive function in mice (Heverin et al., 2015) and increases BACE1 expression via activation of NF‐κB (Marwarha et al., 2013). To investigate the effects of 27‐OHC on BACE1 expression, we treated SH‐SY5Y cells with 27‐OHC. As expected, BACE1 expression was increased by 27‐OHC in a time‐ or dose‐dependent manner (Supporting Information Figure S2A–F). Therefore, we examined whether CO could suppress 27‐OHC‐induced BACE1 expression. As shown in Figure 4a–c, reductions in BACE1 mRNA and protein expression were observed in the CORM2‐and 27‐OHC‐co‐treated cells compared with only 27‐OHC‐treated cells, but the levels of APP mRNA expression did not change (Supporting Information Figure S2G). Because NF‐κB was activated by 27‐OHC treatment in SH‐SY5Y cells (Marwarha et al., 2013), we next performed NF‐κB nuclear translocation and luciferase assay to determine suppressive effects of CO on NF‐κB activation‐mediated BACE1 gene expression. We found that CO reduced nuclear translocation of NF‐κB p65 subunit and NF‐κB reporter activity induced by 27‐OHC (Figure 4d–f). Furthermore, CO decreased the levels of the IκB kinase complex (IKK) and IκBa phosphorylation, which are necessary for NF‐κB activation induced by 27‐OHC (Figure 4g). To examine the effects of CO on BACE1 gene transcription, we transfected SH‐SY5Y cells with BACE1P‐2.5 constructs and then treated with CORM2. CO decreased basal promoter activity of BACE1 as well as the promoter activity induced by 27‐OHC, respectively (Figure 4h,i). In order to test whether CO influences the binding of NF‐κB to BACE1 promoter consistent with the luciferase data, we examined EMSA using oligonucleotide probe including NF‐κB responsive element located in the 5′ upstream of BACE1 promoter (Marwarha et al., 2013). When EMSA was conducted using the BACE1P‐NF‐κB RE probe, dominant DNA–protein complexes as binding of NF‐κB p65 protein to the element of BACE1 promoter were observed, and 27‐OHC increased the intensity of the DNA–protein complexes (Figure 4j, lanes 2). Treatment of CORM2 reduced the DNA–protein complexes, resulting in reduction in the intensity of the NF‐κB p65 shifted bands (Figure 4j, lanes 3 and 4). The DNA–protein complexes were markedly reduced in a dose‐dependent manner by preincubation of the reaction mixture with p65 antibody or addition of 100× unlabeled wild‐type oligonucleotides, but did not reduce the complexes in the reaction mixture pre‐incubated with an isotype control antibody (Figure 4j, lanes 5–7). These results indicate that p65 can specifically interact with the NF‐κB p65‐binding element in the BACE1 promoter regions, and CO decreases this interaction. Moreover, we analyzed chromatin immunoprecipitation (ChIP) assays to confirm the direct binding of p65 to the BACE1 promoter. CORM2 decreased p65 binding to the promoter of BACE1 gene compared with only 27‐OHC‐treated cells (Figure 4k). These results indicate that CO decreases recruitment of NF‐κB p65 to the BACE1 promoter. Thus, these results indicate that CO inhibits BACE1 gene expression through the suppression of NF‐κB signaling.

## 3 DISCUSSION

BACE1 is elevated in brain regions affected by AD, indicating that abnormal BACE1 plays a role in the AD pathogenesis by processing APP to Aβ (Farzan, Schnitzler, Vasilieva, Leung, & Choe, 2000; Johnston et al., 2005; Yang et al., 2003). Metabolic stress gives rise to inflammatory conditions known to greatly increase the incidence of AD (Alzoubi et al., 2013). These observations indicate a potential link between hypoxia‐activated signaling pathways triggering NF‐κB induction and activation of the Aβ generation machinery, which requires up‐regulation of BACE1 level or activity. Therefore, understanding the mechanism of regulation of BACE1 is critical for designing therapeutic strategies for AD.

Herein, we present evidence that CO attenuates the transcriptional expression of BACE1 induced by NF‐κB signaling pathway under basic and oxidative stress or metabolic stress conditions. We explored the molecular pathways and signal transduction mechanisms involved in the CO‐induced attenuation in BACE1 expression and subsequent mitigation in Aβ genesis. Our study indicates that CO negatively regulates BACE1 expression induced by oxidative stress or metabolic stress via inhibition of NF‐κB binding on BACE1 promoter. This is the first report of CO decreasing the BACE1 gene expression.

Previous reports showed that a high‐fat diet containing high cholesterol impairs working memory and induces neuroinflammation characterized by glial activation and cytokine expression (Thirumangalakudi et al., 2008). High levels of circulating cholesterol are associated with high levels of 27‐OHC, and thus, it is likely that hypercholesterolemia is associated with increased flux of 27‐OHC into the brain (Heverin et al., 2005). Moreover, the brains of patients who had died with AD contain significantly increased levels of 27‐OHC (Shafaati et al., 2011). Furthermore, it was reported that 27‐OHC increases the levels of p65 and p50 subunits of the NF‐κB (Marwarha et al., 2013). Indeed, NF‐κB activity has been found to be increased in autopsied brains of AD patients as well as increased NF‐κB immunoreactivity is observed in association with amyloid plaques (Bourne et al., 2007; Chen et al., 2012). These reports indicate that NF‐κB signaling is required for the transcription of BACE1 and neuroinflammation following metabolic stress. It has been previously reported that the BACE1 promoter contains NF‐κB binding elements (Bourne et al., 2007; Chen et al., 2012) and Aβ‐mediated transactivation of the BACE1 promoter involves NF‐κB pathways (Buggia‐Prevot et al., 2008). Herein, we show that CO decreases promoter activity and gene expression of BACE1 as well as β‐CTFs levels under metabolic stress conditions. Our results indicate that reduction in BACE1 by CO is mediated by inhibition of NF‐κB activity in vitro and in vivo. It has been reported that glycogen synthase kinase‐3β (GSK3β) increases the transcriptional expression of BACE1 through NF‐κB, resulting in Aβ production (Ly et al., 2013). Our previous reports showed that CO reduces NF‐κB signaling through GSK3β inactivation (Kim et al., 2013). Therefore, there is the possibility that CO‐mediated GSK3β inactivation might decrease BACE1 expression through negative regulation of NF‐κB.

Moreover, our data show that CO increases the expression level and activity of the master metabolic regulator SIRT1, which subsequently results in the decreased NF‐κB‐mediated BACE1 expression. CO‐induced reduction in BACE1 expression is contingent on SIRT1 activation as the SIRT1 inhibitor abrogates the inhibitory effect of CO on BACE1 expression. Consistently, CO decreases the levels of acetylated‐Lys310 p65 and this reduction is dependent on the suppression of SIRT1 activity. Overexpression of SIRT1 confers neuroprotection from toxic insults and excludes learning deficits in animal models of AD (Kim et al., 2007). SIRT1‐induced deacetylation of the p65 subunit at Lys310 decreases both the DNA binding affinity and induction of NF‐κB‐mediated transcription of target genes (Chen et al., 2005; Yeung et al., 2004). These reports are consistent with our results that CO reduces transcriptional expression of BACE1 through SIRT1‐mediated NF‐κB p65 deacetylation. In addition, SIRT1 deacetylates and accordingly regulates the subcellular localizations and activities of transcription factors that include NF‐κB, p53, PPARγ, and PGC1α. PPARγ and PGC1α are known to attenuate BACE1 transcription (Katsouri, Parr, Bogdanovic, Willem, & Sastre, 2011; Lange‐Dohna et al., 2003; Sastre et al., 2006; Wang et al., 2013). Interestingly, CO has been shown to increase PGC1α expression and PPARγ activity (Kim et al., 2014; Tsoyi et al., 2009). Therefore, further studies are required to verify the involvement of PGC1α and PPARγ in the SIRT1‐dependent modulation of BACE1 expression by CO.

In the present study, we demonstrated that CO reduces NF‐κB‐mediated BACE1 transcription and subsequently mitigates Aβ generation. CO decreases BACE1 protein and mRNA expression levels by attenuating NF‐κB transcriptional and BACE1 promoter activities in a SIRT1‐dependent manner during H_2_O_2_‐mediated oxidative stress. Also, CO reduces BACE1 expression induced by HFC‐mediated metabolic stress. But, it remains still unclear whether metabolic stress by HFC or 27‐OHC could trigger NF‐κB activity by GKS3β activation or SIRT1 inactivation. Thus, we need to further define the role of GSK3 and SIRT1 in regulatory effects of CO on NF‐κB‐mediated BACE1 expression under metabolic stress conditions. Although we have not shown in our results whether SIRT1 or GSK3β is involved in CO‐induced reduction in BACE1 under metabolic stress, it is clearly shown that CO downregulates BACE1 expression through inhibition of NF‐κB activity. Therefore, our data provide a potential therapeutic application of CO in AD pathogenesis induced by metabolic risk factors.

## 4 EXPERIMENTAL PROCEDURES

### 4.1 Cell culture and drug treatment

Human neuroblastoma SH‐SY5Y cells were maintained in DMEM. Media were supplemented with 10% fetal bovine serum (FBS) and a 100 units/ml penicillin/streptomycin mixture (Gibco, Grand Island, NY, USA). Cell lines were incubated at 37°C with 100% humidity in 5% CO2 and passaged using standard cell culture techniques. N2a cells stably expressing human APP Swedish mutation (N2aSwe cells) were donated by Dr. Takeshi Iwatsubo (Department of Neuropathology and Neuroscience, Graduate School of Pharmaceutical Sciences, University of Tokyo) (Saura et al., 2000) and grown in DMEM containing 10% FBS.

### 4.2 Animals

We used 82 C57BL/6 mice and 38 3xTg‐AD mice. Non‐transgenic wild‐type (C57BL/6; non‐Tg) female mice and triple‐transgenic Alzheimer's disease (3xTg‐AD; PS1M146V, APPSwe, and tauP301L) female mice aged 4 weeks were purchased from Hyochang (Daegu, South Korea) and Jackson Laboratory (Bar Harbor, ME, USA), respectively. For HFC experiments, C57BL/6 male mice aged 6 weeks were purchased from Orient Bio (Seongnam, Korea). After acclimatizations over 6 months, non‐Tg and 3xTg‐AD mice were intraperitoneally injected with CORM‐3 or vehicle solution (PBS) at 3 or 10 mg/kg every day for 4 weeks, respectively (4–7 mice/group). High‐fat, high‐cholesterol (HFC) custom diet containing 16% fat and 1.25% cholesterol (D12336; Research Diets, Inc., New Brunswick, NJ, USA) was given to C57BL/6 mice from 5‐month‐old group for 2 months and compared to a control chow diet (7–11 mice/group). Starting at the sixth week, mice inhaled CO (250 ppm) in air (Core Gas Ulsan, Korea) for 2 hr each day for 2 weeks. Mouse studies were performed according to guidelines of the University of Ulsan Animal Care and Use Committee (HTC‐14–030). The mice were maintained under specific pathogen‐free conditions and given access to food and water ad libitum.

### 4.3 Enzyme‐linked immunosorbent assay (ELISA)

Aβ40 and Aβ42 levels were quantified by using an ELISA immunoassay kit (BioLegend, Aβ40: SIG‐38954; Aβ42: SIG‐38956). Brain tissues were lysed in 3 volumes (wt/vol original tissue weight) of TBS+1% Triton X‐100, pelleted for 30 min × 100,000 *g*, and the supernatant recovered and stored. The Aβ40 and Aβ42 levels were quantified in the media (secreted) and cellular homogenates (intracellular) followed by the treatments, the culture medium was collected, supplemented with protease and phosphatase inhibitors cocktail, and centrifuged at 16,000 *g* for 5 min at 4°C. Supernatant was used for the quantification of secreted Aβ40 and Aβ42 levels and was assayed by ELISA according to manufacturer's instruction. To measure the levels of intracellular Aβ40 and Aβ42 in the cellular homogenates, cells were collected by centrifugation at 5,000 *g* and the pellet was resuspended in 70% formic acid. Formic acid samples were then neutralized by adding 1 M Tris base and diluted 1:3 in H_2_O. Intracellular Aβ levels in the cellular homogenates were normalized to total protein content in the samples and quantified by ELISA.

### 4.4 Chromatin immunoprecipitation (ChIP) assay

ChIP assays were analyzed using the EZ‐Magna ChIP™ G KIT (Millipore) according to the manufacturer's instructions. The nuclei were isolated from SH‐SY5Y cells treated with 27‐OHC in the presence or absence of CORM2. Isolated nuclei were sonicated to shear the DNA (approximate average size was 500 bp), and chromatin was immunoprecipitated with rabbit anti‐NF‐κB p65 antibody (Novus, NB100‐2176, 1 μg/μl) or normal rabbit IgG (Millipore, 12‐370, 1 μg/μl). The complexes were collected on Protein G Magnetic Beads and subsequently extracted from the beads. The precipitated DNA was subjected to PCR using primers designed to amplify a 162‐bp fragment (−2,373 to −2,212) of the BACE1 promoter flanking the NF‐κB p65 sites. Total input DNA at a 1:10 dilution was used as positive control for the PCR. The nucleotide sequences of the primers used for ChIP are listed in Supporting Information Table S1. PCR products were resolved on 2% agarose gel in the presence of 1 μg/mL of ethidium bromide.

### 4.5 Statistical analysis

All data were expressed as mean ± *SEM*. The data were analyzed by using GraphPad Prism 5 (GraphPad Software, Inc, La Jolla, CA, USA). Statistical differences were assessed with two‐tailed Student's *t* test, one‐way ANOVA for multiple groups, and two‐way ANOVA for testing two variables across multiple groups with Bonferroni's post‐test. *p* < 0.05 was considered significant.

## CONFLICTS OF INTEREST

The authors have declared no conflict of interest.

## AUTHOR CONTRIBUTIONS

All authors participated in the revision of the manuscript. HJK and YJ contributed to study conception and design. HJK, YJ, YC, and GHP performed experiments and carried out analysis and interpretation of data. HJK, UHK, and HTC drafted the manuscript. All authors commented and approved the manuscript.
