# Supplementary material for: Carbon monoxide attenuates amyloidogenesis via down‐regulation of NF‐κB‐mediated BACE1 gene expression
Source: Aging Cell. 2018 Nov 9;18(1):e12864. doi: 10.1111/acel.12864 (PMC6351829; doi:10.1111/acel.12864)
Supplement: Supplementary file 1 [file ACEL-18-e12864-s001.doc]

**SUPPORTING INFORMATION**

**Carbon monoxide attenuates amyloidogenesis via**

**down-regulation of NF-κB-mediated BACE1 gene expression**

Hyo Jeong Kim^1*^, Yeonsoo Joe^1*^, Yingqing Chen^1^, Gyu Hwan Park^2^,

Uh Hyun Kim^3#^, Hun Taeg Chung^1#^

^1^Meta-Inflammation Research Institute of Basic Research, School of Biological Sciences, University of Ulsan, Ulsan, South Korea. ^2^College of Pharmacy, Research Institute of Pharmaceutical Sciences, Kyungpook National University, Daegu, South Korea. ^3^National Creative Research Laboratory for Ca^2+^ Signaling Network, Chonbuk National University, Medical School, Jeonju, South Korea

**SUPPORTING INFORMATION LISTING**

**Supplemental Methods.**

**Supplemental Table 1.** Primer sequences used for semi-quantitative RT-PCR

**Supplemental Figure S1.** CO reduces BACE1 expression and activity.

**Supplemental Figure S2.** 27-OHC induces BACE1 expression.

**SUPPORTING INFORMATION**

**Supplemental Methods:**

**Reagents**

Tricarbonyl dichlororuthenium (II) dimer (CORM-2), Tricarbonylchloro(glycinato)ruthenium (II) (CORM-3), hydrogen peroxide (H_2_O_2_), and sirtinol and EX527 as a SIRT1 inhibitor, were purchased from Sigma-Aldrich (St. Louis, MO, USA). 27-Hydroxycholesterol (27-OHC) was from Enzo lifesciences (Faimingdale, NY, USA, ENZ-CHM102).

**SDS-PAGE and immunoblotting**

Harvested brain tissues and cells were lysed with mammalian lysis buffer and RIPA buffer containing phosphatase and protease inhibitors. Equal amounts of cell lysates was measured with the BCA protein assay reagent (Pierce Biotechnology, Rockford, IL). Lysates were boiled in sample buffer containing β-mercaptoethanol for 5 min. Proteins were then subjected to SDS-PAGE and transferred to polyvinylidene difluoride membranes (GE healthcare). After blocking with 5% skim milk in PBS, membranes were incubated with appropriate dilutions of antibodies at 4 °C overnight as follows: polyclonal rabbit anti-SIRT1 (Millipore, Temecula, CA, #07-131, 1:3000 dilution), rabbit anti-BACE1 (Abcam, Cambridge, MA, ab108395, 1:1000 dilution), mouse anti-beta amyloid (6E10) (Biolegend, SIG-39320, 1:1000 dilution), rabbit anti-amyloid precursor protein, C-Terminal antibody (Sigma, A8717, 1:1000), rabbit anti-acetylated p65 (Abcam, #52175, 1:1000 dilution), rabbit anti-p65 (Abcam, ab16502, 1:1000 dilution), rabbit anti-phosphorylated IKK (p-IKK) (Cell Signaling, #2697, 1:1000), rabbit anti-IKK (Cell Signaling, #2684, 1:1000), rabbit anti-phosphorylated IκB-α (p-IκB-α) (Santa Cruz, CA, sc-101713, 1:1000), rabbit anti-IκB-α (Santa Cruz, sc-371, 1:1000) and β-actin (Santa Cruz, sc-1616, 1:1000 dilution) were used. Membranes were then washed with 0.05% PBS–Tween 20 and incubated with a 1/5000 dilution of HRP conjugated secondary Abs at room temperature for 1 h. Immunoreactivity was detected using the ECL detection system (GE Healthcare). β-actin was used as an internal control. All band intensities were determined by densitometry (Image J). Basal levels in the untreated sample were set at 1.0, and results are expressed as fold induction over control levels.

**Quantitative real-time RT-PCR (qRT-PCR)**

Total RNA was isolated from cells by using Trizol (Invitrogen Life Technologies, Carlsbad, CA) according to the manufactures protocol. RNA was reverse transcribed to synthesize the first-strand cDNA by using oligo-dT primers (QIAGEN) and M-MLV reverse transcriptase (Promega, Madison, WI) according to the manufacturer's instructions. The cDNA product was subjected to the PCR-based amplification. Semi-quantitative RT-PCR was performed using Taq polymerase (Bioneer, Daejeon, South Korea). Real-time PCR was performed using SYBR Green PCR Master Mix (Applied Biosystems, Foster City, CA) on an ABI 7500 Fast Real-Time PCR System (Applied Biosystems). The mRNA expression data were normalized to GAPDH gene expression. The nucleotide sequences of the primers used for semi-quantitative RT-PCR are listed in Table S1.

**Plasmid preparation, transfection and luciferase assay**

pCAX APP Swe/Ind was a gift from Dennis Selkoe & Tracy Young-Pearse (Addgene plasmid # 30145). A 2802-base pair (bp) fragment of the 5′-flanking region (+227 to -2575) of the BACE1 gene was amplified from the genomic DNA of SH-SY5Y cells by PCR. The amplified fragment was inserted into the KpnI and XhoI sites of the pGL3-basic firefly luciferase vector (Promega). The nucleotide sequences of the primers used for constructs are listed in Table S1. SH-SY5Y cells were transfected with pCAX APP Swe/Ind constructs. After 24 hours, cells were treated with CORM2, and then harvested and assayed. For luciferase assay, SH-SY5Y cells were transfected with the firefly pNF-κB-Luciferase constructs (Stratagene) or BACE1P-2.5 constructs and the renilla luciferase-expressing plasmid (pRL-SV40; Promega, Madison, USA), the latter as a transfection control, were transiently transfected into SH-SY5Y cells using Lipofectamine 2000 reagent (Life Technologies). After 36 h of transfection, cells were harvested and lysed with 100 μl of cell culture lysis buffer (CLB, Promega). Lysates of the transfected cells were analyzed using the dual luciferase assay kit (Promega) according to the manufacturer's protocol. The luciferase activities were measured on a SpectraMax L (Molecular Devices, Sunnyvale, CA). Firefly luciferase activity was normalized to renilla luciferase in each sample. Each experiment was carried out at least three times with triplicate samples.

**Electrophoretic Mobility Shift Assay (EMSA)**

The human BACE1 promoter contains a NF-κB consensus motif 2329 upstream of the transcription start site (Marwarha et al. 2013). The 5’-biotinylated labeled and unlabeled double-stranded oligonucleotide probes that correspond to the NF-κB responsible element (RE) in the human BACE1 promoter regions (-2329 to -2300 of the BACE1 promoter: BACE1P-NF-κB RE probe, 5’-biotin-ggc taa cat ggt gaa ttc ccg tct cca cta-3’) were synthesized by Bioneer (Daejeon, Korea). Nuclear extracts were prepared form SH-SY5Y cells using nuclear/cytosol fractionation kit (BioVision, Mountain View, CA, USA) according to the manufacture’s manual. Binding reactions were carried out for 20 min at room temperature in the presence of 50 ng/ml poly(dI-dC), 0.05% Nonidet P-40, 5 mM MgCl_2_, 10 mM EDTA, and 2.5% glycerol in 1 X binding buffer (LightShift^TM^ Chemiluminescent EMSA kit, Thermo Scientific) using 20 fmol of biotin-end-labeled target DNA and 4 μg of nuclear extract. For competition assay, 100x unlabeled wild-type oligonucleotides (BACE1P-NF-κB RE) were added to the binding reaction. For the supershift assays, 2 μg of anti-NF-κB p65 antibody (Santa Cruz, CA, sc-372x, 2 μg/μl) or normal rabbit IgG (Millipore, 12-370, 2 μg/μl) was added to the binding reaction and incubated overnight at 4°C before adding the biotin-labeled probes. Assays were loaded onto native 5% polyacrylamide gels pre-electrophoresed for 60 min in 0.5 X Tris borate/EDTA and electrophoresed at 100 V before being transferred onto a positively charged nylon membrane (Hybond^TM^-N+) in 0.5 X Tris borate/EDTA at 100 V for 30min. Transferred DNAs were cross-linked to the membrane at 10 mJ/cm2 and detected using horseradish peroxidase-conjugated streptavidin (Lightshift^TM^ chemiluminescent EMSA kit) according to the manufacturer’s instructions.

**Morris water maze (MWM) test**

The Morris water maze was conducted in a circular water pool (1.26 m diameter and 45 cm height, temperature 22 ± 2℃), made white by dissolving non-toxic color. A white circular platform (10 cm diameter and 30 cm height) was placed in one quadrant at the depth of 1 cm from the water surface. During the five subsequent days of training the mice were given three trials per day with the submerged platform in the pool. In each trial, mouse was allowed to swim for 120 s to find the hidden platform and navigate its location based on four different spatial clues surrounding the pool. Once the mouse found the platform, it was permitted to stay on the platform for 10 s. In case the mouse could not find the platform within 120 s, it was navigated to the platform, allowed to stay for 10 s over it, and then returned to the cage. On the sixth day, the hidden platform was removed from the water-maze tank and probe test was performed. Mice were allowed to swim for 90 s and the staying time in the maze quadrant where the platform had previously been located was recorded. The activities of mice were recorded by a video camera linked to a computer installed with EthoVision Pro program (Noldus Information Technology, Wageningen, Netherland) to analyze the time taken to find the platform measured as escape latency.

**Fluorescence microscopy**

Cells were plated on a 4-well Lab-Tek chambered coverglass (Nunc, Thermo Scientific, Waltham, MA). After washing in PBS, cells were fixed with 4% paraformaldehyde in PBS at RT for 10 min, and then were washed with PBS. Cells were permeabilized in 0.2% Triton X-100 for 10 min. Fixed cells were blocked in 10% BSA in PBS for 1 hour at room temperature and incubated in rabbit anti-NF-κB p65 antibody (Novus Biologicals, NB100-2176, 1:100) at 4°C overnight. Cells were then incubated in goat anti-rabbit IgG (H+L) Alexa Fluor 488 secondary antibody (Life Technologies, Carlsbad, CA, R37116, 1:500) at room temperature for 1 hour, and counterstained with 1 μg/ml DAPI (Sigma, D9542) for 20 min. The samples were washed with PBS. GFP was imaged with an Olympus FV1200 confocal microscopy (Olympus, Tokyo, Japan).

**β-secretase (BACE1) Activity Assay**

The activity of β-secretase (BACE1) was performed using the Beta Secretase Activity Assay Kit (Biolegend, K388-100). In brief, Tissue (10 mg) or cells (10 x 106) were extracted with ice-cold BACE1 extraction buffer, incubated on ice for 10 min, and centrifuged (10,000×g) at 4 °C for 5 min. The supernatant was added 2 volumes of saturated 4.32M ammonium sulfate, and incubated on ice for 30 min, and centrifuged (10,000×g) at 4 °C for 10 min. The pellet was resuspended with BACE1 assay buffer. 50 μl of sample was added to each well, followed by 48 μL of BACE1 assay buffer and 2 μl of BACE1 substrate incubated at 37 °C in 1 hr. Activity was measured fluorescently using Synergy HTX Multi-Reader (BioTek; Ex/Em=345/500 nm).

**Immunohistochemistry**

Mice were anesthetized with tribromoethanol and perfused with ice-cold phosphate buffer, followed by 4% paraformaldehyde and transferred to 20% sucrose, overnight. Brains were dehydrated and frozen in optimal cutting temperature (OCT) medium (Tissue-Tek, Torrance, CA) and slide-mounted sections were prepared with a cryostat. For the detection of β-amyloid by immunohistochemistry, SuperPicTureTM Polymer Detection Kit Invitrogen Polymer Detection System (Invitrogen, Frederick, MD, #879663) was used according to the manufacturer's protocols. Briefly, after administrating Peroxo-block solution (Invitrogen, #002015), the sections were incubated with 70% formic acid for 20 min. After washing, the sections were incubated with anti-β-Amyloid (6E10) (Biolegend, 803014, 1:1000 dilution). After overnight incubation, the sections were washed and then incubated with HRP polymer conjugate. Tissue sections were visualized with the substrate DAB chromogen (diaminobenzidine).

**Table S1.** Primer sequences used for semi-quantitative RT-PCR

| **Primer** | **Sequence (5’→3’)** | Assay |
| --- | --- | --- |
| h-SIRT1-F | GCAGATTAGTAGGCGGCTTG | RT-PCR |
| h-SIRT1-R | TCTCCATCAGTCCCAAATCC | RT-PCR |
| Qh-SIRT1-F | TGCTGGCCTAATAGAGTGGCAAAG | qRT-PCR |
| Qh-SIRT1-R | TCTGGCATGTCCCACTATCACTGT | qRT-PCR |
| h-BACE1-F | ATGGGTGAGGTTACCAACCA | RT-PCR |
| h-BACE1-R | TCCATGTCCAAGGTGACAAA | RT-PCR |
| Qh-BACE1-F | CGTTTGCCCAAGAAAGTGTT | qRT-PCR |
| Qh-BACE1-R | ACACCAGCTGCTCTCCTAGC | qRT-PCR |
| h-GAPDH-F | AGG CCG GTG CTG AGT ATG TC | RT-PCR |
| h-GAPDH-R | TGC CTG CTT CAC CAC CTT CT | RT-PCR |
| Qh-GAPDH-F | CAA TGA CCC CTT CAT TGA CCT C | qRT-PCR |
| Qh-GAPDH-R | AGC ATC GCC CCA CTT GAT T | qRT-PCR |
| Q-mGAPDH-F | GGGAAGCCCATCACCATCT | qRT-PCR |
| Q-mGAPDH-R | CGGCCTCACCCCATTTG | qRT-PCR |
| m-BACE1-F | CAG TGG AAG GTC CGT TTG TT | RT-PCR |
| m-BACE1-R | CTA AAG GAT GCT GGG CAG AG | RT-PCR |
| Qm-BACE1-F | GCTTGCACCTGTAGGACACA | qRT-PCR |
| Qm-BACE1-R | CTAAAGGATGCTGGGCAGAG | qRT-PCR |
| Qm-TNF-α-F | AGA CCC TCA CAC TCA GAT CAT CTT C | qRT-PCR |
| Qm-TNF-α-R | TTG CTA CGA CGT GGG CTA CA | qRT-PCR |
| Qm-IL6-F | CGA TGA TGC ACT TGC AGA AA | qRT-PCR |
| Qm-IL6-R | TGG AAA TTG GGG TAG GAA GG | qRT-PCR |
| Qm-IL-1β-F | TCGCTCAGGGTCACAAGAAA | qRT-PCR |
| Qm-IL-1β-R | ATCAGAGGCAAGGAGGAAACAC | qRT-PCR |
| h-BACE1prom-2.5U | CGGGGTACCCCTAATAGCTGACCTTTATTGAG | Luciferase assay |
| h-BACE1prom+227D | CCGCTCGAGCGGCGGCTGTCAAAGCCAAAAGG | Luciferase assay |
| hBACE1-ChIP-F | TGAGGCAGGCAGATAACTTG | ChIP assay |
| hBACE1-ChIP-R | GCCTCCTCAAGCGATTCTC | ChIP assay |

**Table S1.** Primer sequences used for semi-quantitative RT-PCR *(Continued)*

| **Primer** | **Sequence (5’→3’)** | Assay |
| --- | --- | --- |
| Qh-APP-F | TTTGGCACTGCTCCTGCT | qRT-PCR |
| Qh-APP-R | CCACAGAACATGGCAATCTG | qRT-PCR |
| Qm-APP-F | GCTGGCCTGCTGGCTGAACC | qRT-PCR |
| Qm-APP-R | GGCGACGGTGTGCCAGTGAA | qRT-PCR |

**Supplemental Figure S1. CO reduces Aβ plaque, BACE1 expression and activity.** (A) Representative brain sections of WT, vehicle- or CORM-treated 3xTg mice, respectively. Amyloid plaques were stained with Aβ specific monoclonal antibody 6E10. Scale bar = 50 μm. (B) HFC diets fed mice were inhaled with air or CO. The levels of APP mRNA expression were examined by qRT-PCR. (C) N2aswe cells were treated with CORM2 at the indicated doses for 6 hours. Protein levels of β-CTF and β-actin were performed by immunoblotting. β-CTFs were detected with 6E10 antibody. (D-H) SH-SY5Y cells were incubated with 200 μM of H_2_O_2_ for 3 h in the presence or absence of CORM2 at the indicated doses. The levels of SIRT1 (D and E) and BACE1 (F and G) mRNA expressions were measured by semi-quantitative RT- PCR (D and F) and qRT-PCR (E and G). H, The levels of APP mRNA expression was examined by qRT-PCR. (I) SH-SY5Y cells were transfected with NF-κB responsive element reporter constructs (NF-κB-Luc) and pRL-SV40 renilla constructs for 48 h. Cells were pretreated with CORM2 at the indicated doses, and stimulated with 200 μM of H_2_O_2_. After 6 h, luciferase activity was measured. The levels of firefly luciferase activity were normalized to renilla luciferase activity. Data were shown as mean ± SEM of three independent experiments. **P*< 0.05, ***P*< 0.01. ns, not significant.

**Supplemental Figure S2. 27-OHC induces BACE1 expression.** (A and B) SH-SY5Y cells were treated with 27-OHC at the indicated doses for 24 h. (C-F) SH-SY5Y cells were treated with 5 μM (C and D) and 10 μM (E and F) 27-OHC for the indicated times. The levels of BACE1 mRNA expression were examined by semi-quantitative RT-PCR (C and E) and qRT-PCR (D and F). (G) SH-SY5Y cells were incubated with 5 μM of 27-OHC for 12 h in the presence or absence of CORM2 at the indicated doses. APP mRNA expression was examined by qRT-PCR. Data wer shown as mean ± SEM of three independent experiments. **P*< 0.05, ***P*< 0.01. ****P*< 0.001. ns, not significant.


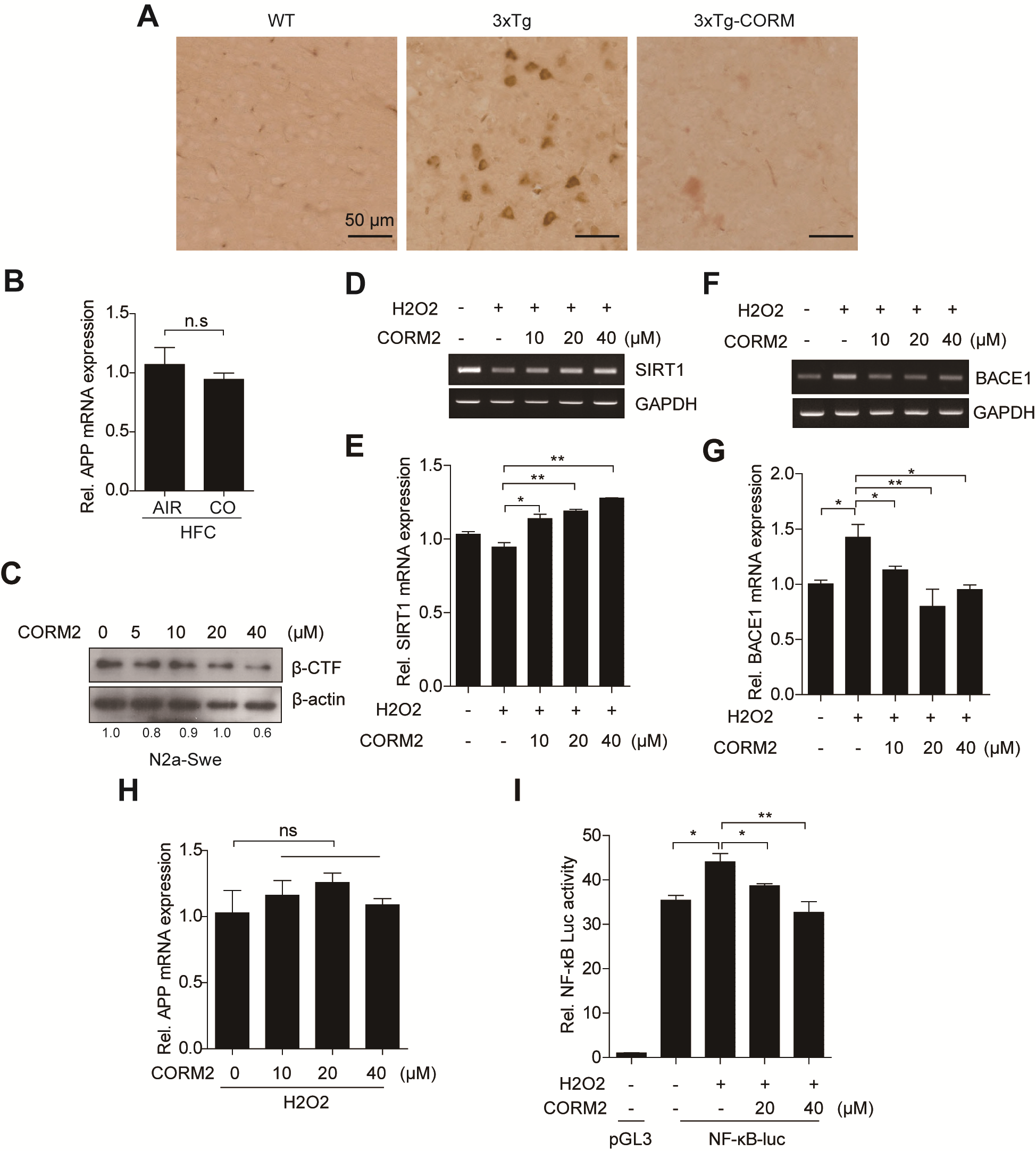


**Supplemental Figure S1**


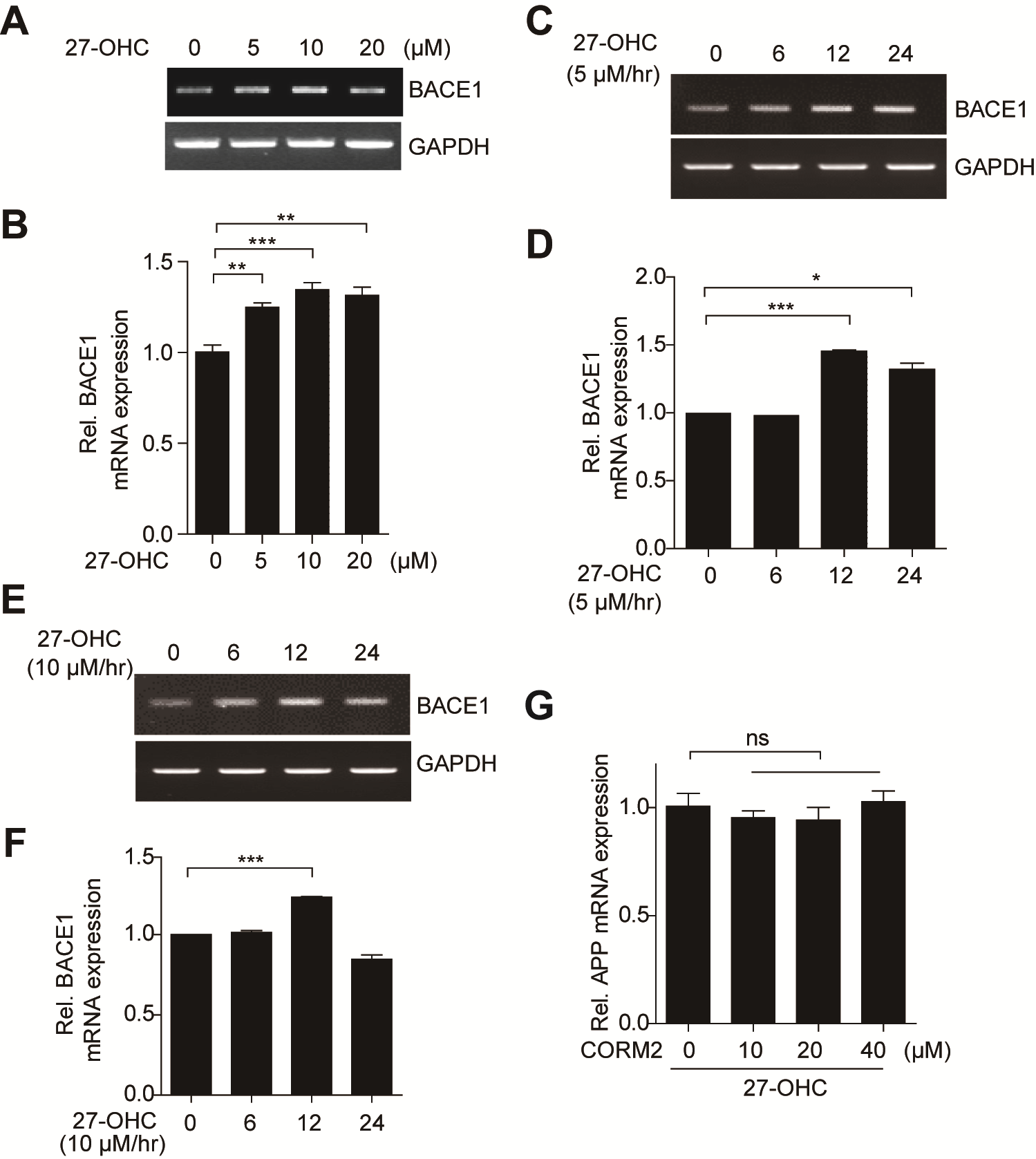


**Supplemental Figure S2**
